# Probabilities of ICU admission and hospital discharge according to patient characteristics in the designated COVID-19 hospital of Kuwait

**DOI:** 10.1186/s12889-021-10759-z

**Published:** 2021-04-26

**Authors:** Dimitra-Kleio Kipourou, Clémence Leyrat, Nourah Alsheridah, Sulaiman Almazeedi, Sarah Al-Youha, Mohammad H. Jamal, Mohannad Al-Haddad, Salman Al-Sabah, Bernard Rachet, Aurélien Belot

**Affiliations:** 1 Inequalities in Cancer Outcomes Network, Department of Non-Communicable Disease Epidemiology, Faculty of Epidemiology and Population Health, London School of Hygiene & Tropical Medicine, London, UK; 2 Department of Medical Statistics, Faculty of Epidemiology and Population Health, London School of Hygiene & Tropical Medicine, Keppel Street, London, UK; 3 COVID-19 Research Group, Jaber Al-Ahmad Al-Sabah Hospital, Kuwait City, Kuwait

**Keywords:** Competing risks, COVID-19, Probability of ICU admission

## Abstract

**Background:**

Subsequent epidemic waves have already emerged in many countries and in the absence of highly effective preventive and curative options, the role of patient characteristics on the development of outcomes needs to be thoroughly examined, especially in middle-east countries where such epidemiological studies are lacking. There is a huge pressure on the hospital services and in particular, on the Intensive Care Units (ICU). Describing the need for critical care as well as the chance of being discharged from hospital according to patient characteristics, is essential for a more efficient hospital management. The objective of this study is to describe the probabilities of admission to the ICU and the probabilities of hospital discharge among positive COVID-19 patients according to demographics and comorbidities recorded at hospital admission.

**Methods:**

A prospective cohort study of all patients with COVID-19 found in the Electronic Medical Records of Jaber Al-Ahmad Al-Sabah Hospital in Kuwait was conducted. The study included 3995 individuals (symptomatic and asymptomatic) of all ages who tested positive from February 24th to May 27th, 2020, out of which 315 were treated in the ICU and 3619 were discharged including those who were transferred to a different healthcare unit without having previously entered the ICU. A competing risk analysis considering two events, namely, ICU admission and hospital discharge using flexible hazard models was performed to describe the association between event-specific probabilities and patient characteristics.

**Results:**

Results showed that being male, increasing age and comorbidities such as chronic kidney disease (CKD), asthma or chronic obstructive pulmonary disease and weakened immune system increased the risk of ICU admission within 10 days of entering the hospital. CKD and weakened immune system decreased the probabilities of discharge in both females and males however, the age-related pattern differed by gender. Diabetes, which was the most prevalent comorbid condition, had only a moderate impact on both probabilities (18% overall) in contrast to CKD which had the largest effect, but presented only in 7% of those admitted to ICU and in 1% of those who got discharged. For instance, within 5 days a 50-year-old male had 19% (95% C.I.: [15,23]) probability of entering the ICU if he had none of these comorbidities, yet this risk jumped to 31% (95% C.I.: [20,46]) if he had also CKD, and to 27% in the presence of asthma/COPD (95% C.I.: [19,36]) or of weakened immune system (95% C.I.: [16,42]).

**Conclusions:**

This study provides useful insight in describing the probabilities of ICU admission and hospital discharge according to age, gender, and comorbidities among confirmed COVID-19 cases in Kuwait. A web-tool is also provided to allow the user to estimate these probabilities for any combination of these covariates. These probabilities enable deeper understanding of the hospital demand according to patient characteristics which is essential to hospital management and useful for developing a vaccination strategy.

**Supplementary Information:**

The online version contains supplementary material available at (10.1186/s12889-021-10759-z).

## Introduction

A cluster of pneumonia cases was reported in Wuhan, China, in the last weeks of 2019 [1]. The virus responsible for this ensemble of symptoms, subsequently named COVID-19 [2, 3], was quickly identified, sequenced and called the Severe Acute Respiratory Syndrome Coronavirus-2 (SARS-COV-2) [4]. The responsible virus was characterised as easily contagious and soon the epidemic quickly spread first within China and then across the rest of the world. The World Health Organization (WHO) declared that the outbreak was of international concern at the end of January 2020 and announced the pandemic in March 2020 [5]. Soon it became apparent that the virus could cause severe forms of the disease, sometimes lethal, in particular for individuals of older ages or with comorbid conditions [3]. Given these characteristics, the human cost of this pandemic has continued to mount with to date over 60 million cases and 1.4 million deaths from COVID-19 [6].

The onset of COVID-19 outbreak in the Gulf Cooperation Council (GCC) countries was evidenced between February 2020-March 2020 [7, 8]. At the end of January, the first known incident case of COVID-19 in GCC was reported by the United Arab Emirates, soon followed by early COVID-19 case reports made by Bahrain, Kuwait, Qatar, and Oman in late February 2020 [8]. The government of the state of Kuwait instructed the repatriation of all their citizens and relatives just before the surge of cases in Iran [9].

Worldwide, lockdown policies have been applied to most countries in an effort to mitigate the widespread of the virus, flatten the epidemic curve, and reduce the burden of the pandemic on the health system [3]. Kuwait in particular, promptly applied aggressive controls to surround and contain the disease; all international flights from Kuwait airport were suspended, land borders with neighbouring countries were closed, as well as, schools, universities, governmental offices and businesses were shut [8, 10]. These measures were accompanied by a partial curfew and further restrictions on geographical areas of high-risk of community transmission [10]. Other strict measures were also enforced in order to contain the pathogen from spreading, including the use of the reverse transcriptase-polymerase chain reaction (RT-PCR) test for all arrivals and their extended families along with the susceptible individuals who came in contact with confirmed COVID-19 cases [9]. The screening also covered high-risk residential areas including those with large number of migrant workers who are living in big households within a minimal space [9, 11]. Furthermore, from the 24th of February 2020 until the 12th of May 2020, all known cases, symptomatic or asymptomatic, were sent to a single designated COVID-19 centre, the Jaber Al-Ahmad Al-Sabah hospital. As such, Jaber hospital was used both as a quarantine and treatment facility by the Government of Kuwait during that period, and solely as a treatment centre afterwards.

Subsequent epidemic waves have already emerged in many countries and in the absence of highly effective preventive and curative options, there is a need for detailed epidemiological examination of the role of patients characteristics on the development of outcomes. Here, we aim to describe how the patients characteristics (demographics and comorbidities) are associated with the probabilities of admission to an Intensive Care Unit (ICU) and the probabilities of hospital discharge among the cohort of patients admitted into Jaber Hospital in Kuwait.

## Methods

### Study design and patient recruitment

This is a prospective cohort study including all confirmed COVID-19 cases in Kuwait admitted to Jaber Hospital between 24 of February 2020 and 27 of May 2020. All these cases, either symptomatic or asymptomatic, were confirmed COVID-19 cases, based on a real-time reverse-transcriptase-polymerase chain-reaction (RT-PCR) assay of nasopharyngeal swab specimens. Patients with equivocal or negative results were excluded from the analysis.

Data on all confirmed COVID-19 cases, extracted from Electronic Medical Records (EMR) of Jaber Hospital, contained information on their demographic (age, sex, and residency) and anthropometric characteristics, their medical history (including comorbidities), and clinical presentation (signs, symptoms, laboratory investigations). These clinical data were obtained as part of the routine clinical practice of the hospital and not specifically for this study. All data were de-identified to ensure confidentiality and subsequent checks have been performed to ensure accuracy of the data and minimize data entry error. Ethical approval for this cohort and study was obtained from the Kuwait Ministry of Health’s Ethical Review Committee (ERC) (No. 2020/1402) and the LHSTM Ethical Committee (No 22676).

### Outcomes

The patients could enter the ICU, get discharged from the hospital, transferred to a different healthcare unit, or die.

Patients are assessed by the rapid response team based on the presence of 3 out of 7 of the following criteria:

- Age greater than 60 years;
- Heart rate greater than 100 beats per minute;
- Systolic blood pressure lower than 90mmHg or Mean arterial pressure lower than 65mmHg;
- Temperature greater than 38.1 C;
- Respiratory Rate greater than 26 breaths per minute;
- Saturation lower than 92% on arterial blood gas or any saturation while the patient on supplemental oxygen;
- Any pulmonary infiltrates not considered chronic changes (chronic obstructive pulmonary disease including emphysema, any interstitial or fibrotic lung disease)

and are admitted to ICU if they are deemed likely to require ventilatory or hemodynamic support. Patients with a significant past medical history (hypertension, diabetes, ischemic heart disease, chronic renal failure, the immunocompromised or any other comorbidity) are considered of higher risk patients and may have a lower threshold for ICU admission.

We considered two competing events of interest, ICU admission and hospital discharge (including those who were transferred to a different care unit), on the basis of “whichever event occurred first”. Individuals who had not experienced any of those events were regarded as censored. We defined two quantities, the event-specific hazard and the cumulative probability of experiencing one of these events as a function of time since admission in Jaber Hospital due to COVID-19.

### Statistical analysis

We estimated the event-specific hazards using flexible hazard regression models [12] separately for ICU admission and discharge and further combined these results in order to estimate the event-specific probabilities of each event. The splines embedded in the flexible models are used for modelling the baseline hazard function and potential non-linear and time-varying associations between the outcome and the covariables. With these models we are also able to estimate the cumulative probabilities not only at population or group level, but also at individual (i.e., covariate-specific) level according to patient characteristics at hospital admission (i.e. age, gender, comorbidities).

Some socio-demographic and clinical characteristics were retained a priori in the analysis after the guidelines from the European Centre for Disease Prevention and Control and other recent findings. They included gender, age at admission, and the presence of various comorbidities at admission. To avoid small numbers of cases within strata, some of these comorbidities were logically grouped together, namely, cardiovascular diseases (CVD) with hypertension (HT), severe asthma with chronic obstructive pulmonary disease (COPD), and cancer with immunodeficiency and immunosuppression to represent those with weakened immune system. We also included diabetes mellitus (DM) and chronic kidney disease (CKD), as well as accounted for a combination of comorbidities gathering conditions that were not covered previously, namely dyslipidemia, hepatitis, hypothyroidism, and recent surgery (during the past 30 days) to which we refer here as ‘other’. All variables were binary except the continuous variable age. BMI and smoking status were not considered because of their high proportion of missing values. The rest of the variables used in this analysis had no missing values.

A new health policy was introduced by the Ministry of Health as of 12 May 2020 in Kuwait, instructing that the hospital should operate only as a treatment facility hence, limiting the hospital admission only to severe cases (i.e., high-risk patients or those with pneumonia). To account for the new policy, the models included, in addition to the a priori covariables, a binary variable describing the date of hospital admission split into two time windows (before or after the 12th May of 2020).

Additionally, we examined the need for interactions between age and other covariables to account for the possibility that an association between the rest of the covariables and the outcome varies with age. We run several models and chose the final based on the Bayesian Information Criterion which is a simple and reliable model selection approach for descriptive purposes [13]. The performance of the final models was assessed with a graph where population non-parametric estimated probabilities were plotted against their model-based equivalents (see Section 3 of Appendix).

The final model for the ICU admission included the following covariables: age, gender, hospital admission period, DM, CKD, CVD/HT, asthma/COPD, weakened immune system and ‘other’ comorbidities. Conversely, the final model related to hospital discharge considered additionally to the aforementioned covariables, an interaction between age and gender. In both, age was modelled with a quadratic spline with one knot at 41 years and the baseline hazard was specified with a cubic B-spline with one knot located close to their median time-to-event.

Lastly, we combined the results from the two event-specific models in order to estimate the cumulative probabilities of ICU admission and hospital discharge [14]. We plotted the probabilities of each event according to various covariable patterns in order to explore the impact of each covariable on the event-specific risk along the time since hospital admission. We also built a user-friendly web-based tool which enables for instance a clinician who would like to display such probabilities (all derived from the parameters of the final models) for various combinations of factors of interest https://icon.lshtm.ac.uk/cumulative-probabilities/.

Additional technical details on the method used to compute these results can be found in the Appendix (see Section 1). As a sensitivity analysis, we also repeated all the analyses using Cox models instead of flexible hazard models. Since the event-specific hazard ratios were very similar, only those derived from the flexible models were presented. We also conducted a further analysis separately on Kuwaiti and non-Kuwaiti to briefly examine how cohort effects vary with nationality (see Section 4 of Appendix). Lastly, we also conducted a sensitivity analysis to check if the inclusion of the 6 dead people to the censoring group are affecting the results. All data management and statistical analyses were performed using R v4.0.2 software.

## Results

### Patient characteristics

Table 1 describes the baseline characteristics of the whole cohort used for the analysis (including censored observations), separately for each event of interest.

**Table 1:** Cohort description of patient characteristics at hospital admission, by event type

		Number of	Number of	Number of
		individuals	individuals who	individuals who
		(column %)	entered the ICU	got discharged
			(column %)	(column %)
**Total**		**3995 (100)**	**315 (8**^**∗**^**)**	**3619 (90.5**^**∗**^**)**
Age group, years	0-17	291 (7.28)	2 (0.63)	288 (7.96)
	18-39	1648 (41.25)	26 (8.25)	1600 (44.21)
	40-49	809 (20.25)	75 (23.81)	721 (19.92)
	50-59	652 (16.32)	92 (29.21)	548 (15.14)
	60-69	418 (10.46)	75 (23.81)	333 (9.2)
	70-79	140 (3.5)	36 (11.43)	101 (2.79)
	80+	37 (0.93)	9 (2.86)	28 (0.77)
Gender	Male	2814 (70.44)	265 (84.13)	2500 (69.08)
	Female	1181 (29.56)	50 (15.87)	1119 (30.92)
BMI	<18.5	92 (2.3)	1 (0.32)	90 (2.49)
	18.5-24.9	688 (17.22)	35 (11.11)	642 (17.74)
	25-29.9	836 (20.93)	63 (20)	757 (20.92)
	30-34.9 (obese class I)	402 (10.06)	44 (13.97)	354 (9.78)
	35-39.9 (obese class II)	159 (3.98)	19 (6.03)	138 (3.81)
	≥40 (obese class III)	81 (2.03)	9 (2.86)	72 (1.99)
	Missing	1737 (43.48)	144 (45.71)	1566 (43.27)
Smoker	No	2129 (53.29)	157 (49.84)	1927 (53.25)
	Yes	140 (3.5)	8 (2.54)	127 (3.51)
	Missing	1726 (43.2)	150 (47.62)	1565 (43.24)
Nationality	Non Kuwaiti	2356 (58.97)	217 (68.89)	2103 (58.11)
	Kuwaiti	1639 (41.03)	98 (31.11)	1516 (41.89)
Hypertension		778 (19.47)	138 (43.81)	628 (17.35)
Diabetes		730 (18.27)	130 (41.27)	591 (16.33)
CVD		194 (4.86)	54 (17.14)	134 (3.7)
CKD		72 (1.8)	20 (6.35)	49 (1.35)
COPD		17 (0.43)	4 (1.27)	12 (0.33)
Cancer		56 (1.4)	9 (2.86)	44 (1.22)
Asthma		235 (5.88)	31 (9.84)	200 (5.53)
Immunodeficiency		11 (0.28)	2 (0.63)	8 (0.22)
Immunosuppression		14 (0.35)	4 (1.27)	10 (0.28)
Admission date	24/02/20 to 11/05/20	3243 (81.18)	199 (63.17)	2998 (82.84)
	12/05/20 to 27/05/20	752 (18.82)	116 (36.83)	621 (17.16)

Characteristics of censored individuals are not reported separately but included in the total

^*^row percentage (%); 1.5% are censored

The hospital encompasses a diverse patient population concerning demographic characteristics and clinical presentations. During a follow-up period of up to 124 days, 3995 individuals were admitted to the hospital between 24 February to 27 May in 2020, out of which 315 (8%) had to be treated in the ICU and 3619 (90.5%) were able to exit the hospital facility or be transferred to a different health care unit without previously entering the ICU. Among the remainder (1.5%), 55 were still hospitalized outside ICU (with or without treatment) and 6 died before entering the ICU.

Those entering the ICU were mostly between 40 and 69 years, 84% males, 69% being non-Kuwaiti. In comparison, the discharge group was slightly younger, with 69% males and 58% non-Kuwaiti. Over 40% of the ICU group had hypertension and diabetes, which were observed in 17% of the discharge group. Information on BMI and smoking status was missing in about 43% of the cohort. The proportion of BMI at 25 and over was still the majority in the ICU group, as was the proportion of non-smokers in both groups. The comorbidity burden was generally lower in the discharge group. In both groups, the majority of admission occurred in the first time window between 24 Feb 2020 and 11 May 2020, accounting for 63% of ICU admissions and 83% of hospital discharges.

### Description of events

The majority of the ICU admissions occurred in the first days following hospital admission, with almost 90% happening within 5 days, whereas half of the discharges took place in the first 10 days post hospital admission. The timing of events was also reflected on the event-specific population cumulative probabilities for ICU admission (CPr _ICU_(*t*)) and hospital discharge (CPr _Dis_(*t*)) through different times since hospital admission (see Section 2 of Appendix). CPr _ICU_(*t*) was already 4.6% (95%C.I.: [4,5.3]) on admission day, gradually moving to 7.1% (95%C.I.: [6.4,8]) within 5 days, and reaching a plateau at 7.9% (95%C.I.: [7.1,8.8]) a few days later. As low as 2.7% (95%C.I.: [2.2,3.2]) on admission day, CPr _Dis_(*t*) rapidly progressed to 48.6% (95%C.I.: [47,50]) within 10 days after hospital admission reaching finally a maximum at 92% (95%C.I.: [91,93]) within 30 days after hospital admission.

### Results derived from the models

**Fig. 1:**
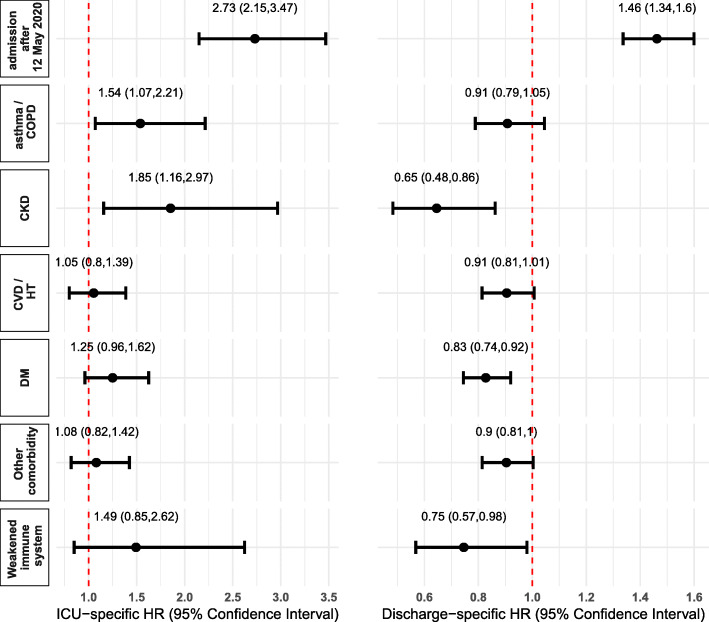
ICU-specific (left) and discharge-specific (right) hazard ratios with 95% confidence intervals. Asthma/COPD: asthma or chronic obstructive pulmonary disease, CKD: chronic kidney disease, CVD/HT: cardiovascular diseases or hypertension, DM: diabetes mellitus

**Fig. 2:**
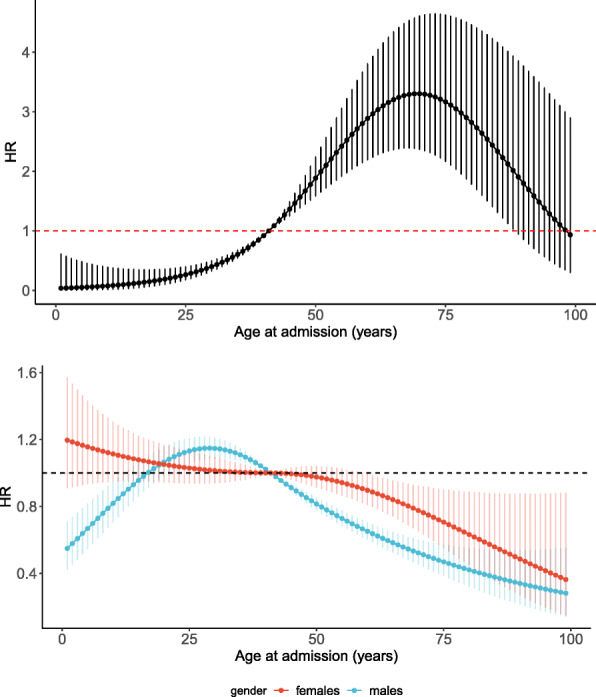
ICU-specific (upper panel) and discharge- and gender-specific (lower panel) hazard ratios (HRs) with 95% confidence intervals for age at admission. HRs are adjusted for the other covariates and reference age is 41

**Event-specific hazards** Figure 1 displays the event-specific hazard ratios of ICU admission and hospital discharge associated with selected key factors of interest. Following the authority instruction, only patients with severe forms of COVID-19 were admitted to Jaber Hospital from the 12th of May 2020 onwards, as clearly indicated by their hazard of being transferred to ICU which was multiplied by 2.7 in comparison to patients in the first period after adjusting on other covariates. This may have not reflected yet in lower hazard of discharge because of the short follow-up of the second-period patients. Younger patients experienced a lower hazard of entering the ICU compared to their older peers, after adjusting on gender, time of admission and comorbidities (Fig. 2-upper panel). The peak was reached at age around 70 with a hazard ratio equal to 3.3. The decrease of the ICU-specific HR for patients older than 80 should be interpreted with caution given the small number of patients observed in these age group (see Table 1). The hazard of ICU admission was 70% lower for females than for males. The picture was slightly more complex for hospital discharge as the age-related hazards varied by gender (Fig. 2-lower panel). Among women, the overall pattern showed a smooth decrease in the discharge-specific hazard with increasing age, with a hazard ratio stable around 1 between 30 and 50 years of age. By contrast, such a pattern was not observed among males where the youngest male patients (less than 20 years old) experienced a lower discharge-specific hazard in comparison to middle-aged male patients. The remaining event-specific hazards were generally consistent with higher hazards of ICU associated with lower hazards of discharge (Fig. 1). Presence of asthma/COPD and CKD increased significantly the ICU-specific hazard, with corresponding lower discharge-specific hazards (though not significant for asthma/COPD). Discharge-specific hazards were also significantly lower in the presence of DM and weakened immune system, with higher but non-significant ICU-specific hazards.

**Event-specific cumulative probabilities** From this section onwards, we will refer to cumulative probabilities simply as probabilities for convenience.

**Fig. 3:**
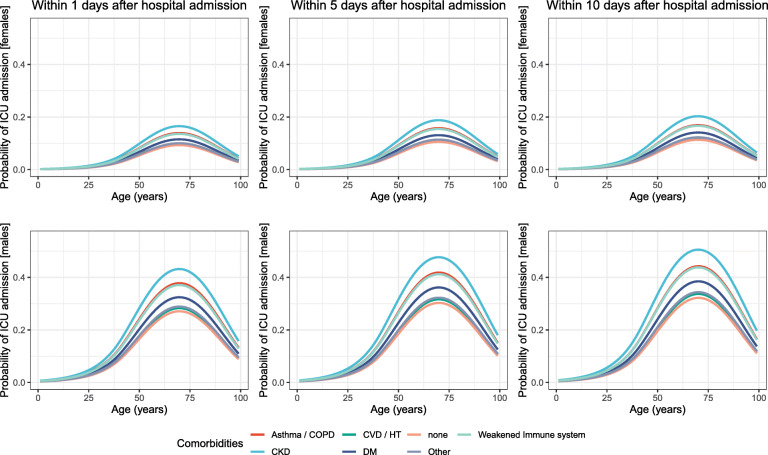
Cumulative probability of ICU admission according to baseline characteristics (age, gender and existence of comorbidities) for females (upper row) and males (lower row) predicted within 1, 5 and 10 days post hospital admission. Comorbidities include: chronic kidney disease (CKD), cardiovascular diseases (CVD) or hypertension (HT), asthma or chronic obstructive pulmonary disease (COPD), weakened immune system and other comorbidities such as dyslipidemia, hepatitis, hypothiroidism, recent surgery (during the past 30 days) etc

**Fig. 4:**
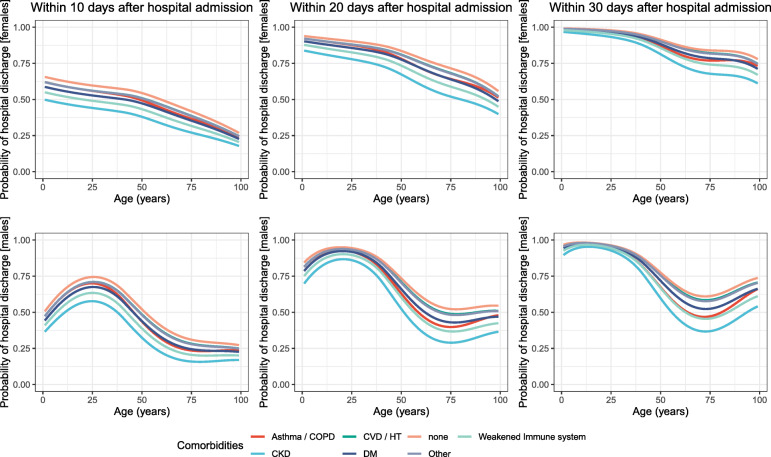
Cumulative probability of hospital discharge according to baseline characteristics (age, gender and existence of comorbidities) for females (upper row) and males (lower row) predicted within 10, 20 and 30 days post hospital admission. Comorbidities include: chronic kidney disease (CKD), cardiovascular diseases (CVD) or hypertension (HT), asthma or chronic obstructive pulmonary disease (COPD), weakened immune system and other comorbidities such as dyslipidemia, hepatitis, hypothiroidism, recent surgery (during the past 30 days) etc

Figures 3 and 4 show the gender-specific probabilities (i) of ICU admission and (ii) hospital discharge according to age and various comorbid conditions. Probabilities are computed and displayed at different times since hospital admission for each event. Results were illustrated for the period after 12 May 2020 using the flexible regression models described in the Methods section whereas the equivalent for the previous period can be found in Section 5 of Appendix. Probabilities presented in both Fig 3 and 4 account for the existence of a single comorbidity at a time. Our models however enable the estimation of these probabilities for any combinations of age, gender, time period and comorbid conditions. To display such probabilities (all derived from the parameters of the final models) for various combinations of factors of interest we refer the reader to our web tool found at https://icon.lshtm.ac.uk/cumulative-probabilities/.

**Probabilities of ICU admission** The probability of ICU admission varied dramatically by age, following a similar pattern in both females and males, and across the various comorbidities, was higher in men and increased with some comorbidities (Fig. 3). Overall, ICU-risk increased with age and was highest at around age 70. Among individuals with none of the comorbidities, the probability to enter the ICU went as high as 9% for females and 27% for males only 1 day post hospital admission. Within 5 days, these probabilities increased to 11% and 30% before stabilising in 10 days at 11% and 31% for females and males, respectively. The presence of any of the comorbidities studied reinforced the risk of ICU admission with the most remarkable increase noticed in the case of CKD, followed by asthma/COPD and weakened immune system. For instance, within 5 days a 50 year old male had 19% (95% C.I.: [15,23]) probability of entering the ICU if he had none of these comorbidities, yet this risk jumped to 31% (95% C.I.: [20,46]) if he had also CKD, and to 27% in the presence of asthma/COPD (95% C.I.: [19,36]) or of weakened immune system (95% C.I.: [16,42]). The gender gap was so large that these probabilities were still higher than those of a female 72 old within 10 days after admission, with or without comorbidity. Lastly, whilst the time-varying probabilities of ICU admission for the individuals with DM were clearly higher than those of individuals with none of the comorbidities, presence of CVD/HT or the comorbidities categorised as ‘other’ was marginally associated with an increase in the probability of being admitted to the ICU for both males and females at all ages.

**Probabilities of hospital discharge** The probabilities of discharge differed considerably between females and males (Fig. 4). With respect to age, there is an almost monotonic decrease in female probabilities (irrespective of the time since hospital admission) which is not true for males whose pattern is more complex. More specifically, probabilities increased in males at all times until 25 and later decreased until 75. This was followed by a subsequent increase which was only minimal within 10 days, more profound within 20 days and quite steep within 30 days. Although this is not always the case for previous times, in 30 days females did much better than males at all ages regardless of their comorbidity status.

Nevertheless, despite the obvious differences, some similar patterns between females and males do exist. Individuals with CKD had the lowest probability of discharge irrespective of age and time since admission, followed by those with weakened immune system. Conversely, the probability of discharge among patients with CVD/HT or ‘other’ comorbidities was similar to that of patients with none of the comorbidities. Presence of comorbidities impacted the event-specific probabilities of all ages within 10 or 20 days post hospital admission yet this effect was only minimal in younger individuals after 30 days.

## Discussion

As additional waves of the COVID-19 pandemic are likely to occur before a large-enough vaccination coverage of the populations is reached, quantitative epidemiological indicators are increasingly needed to provide a clearer picture of the disease progression and to describe how these indicators vary according to the main patient baseline characteristics. A detailed description of the cumulative probabilities/risks of (i) Intensive Care Unit (ICU) admission and (ii) hospital discharge, according to prognostic factors already identified (e.g. age, comorbidities etc.), is of great value to improve the management of the burden that the pandemic waves cause on the healthcare systems and societies. Such indicators are even more crucial in middle-east region where this epidemiological evidence is lacking.

Our study suggests that being male, increasing age and the comorbidities CKD, asthma/COPD and weakened immune system increased the risk of ICU admission within the first 10 days after hospital admission. Equally, CKD and weakened immune system decreased considerably the probabilities of discharge in both genders. Yet with respect to age, conclusions widely varied between females and males. Probabilities in females were deteriorating steadily with age whereas probabilities for males were progressing in a non-monotonic fashion, allowing for both increase/decrease with age. In both events of ICU and discharge, DM had only a moderate impact on both probabilities despite being the most prevalent comorbid condition (18% overall) in contrast to CKD which had the largest effect, but presented only in 7% of those admitted to ICU and in 1% of those who got discharged.

The strengths of our study are mostly attributed to its analytical approach. The estimation of the probabilities of ICU admission and hospital discharge accounted for their temporal component, i.e. the time since hospital admission. We also used flexible event-specific hazard regression models which allow non-linear functions to be modelled and the events to be analysed separately. The selection of key demographic and clinical characteristics (including a range of comorbidities) was made a priori. Furthermore, the different sensitivity analyses performed to assess some assumptions did not modify the study conclusions. Yet, the study could be extended further to accommodate more states into its design including the transition between ICU and discharge or adding death as an additional event. Analysing the complete pathway would provide a clearer guidance on the COVID-19 treatment needs and practices. However, only six individuals in this cohort died prior to ICU/discharge hence, modelling those separately was not an option and they were censored. Excluding those six from the analysis hardly modified the results. Lastly, two potential risk factors (i.e. BMI and smoking) could not be considered due to the large amount of missing data. Overcoming these issues and including them in the analysis by applying appropriate methodologies for missing data will be also part of future work.

Our results are based on a single centre, which could affect their generalisability, but it is also worth highlighting the great homogeneity in data collection and that Jaber Hospital captured the vast majority of COVID-19 patients in Kuwait at that time, regardless of the severity of the disease. Therefore, unlike many studies in hospital settings this one also included asymptomatic cases for its most part, which reduces the risk of collider bias because of conditioning on admission [15]. These, combined with the analytical components, may explain some of the differences between the literature and our results.

Most studies in the Middle East region focused on risk factors of mortality [16–19] among COVID-19 patients and, to our knowledge, very little (including a few papers from subsequent parts of the Jaber Hospital cohort) has been published on factors associated with the risk of ICU admission. More globally, an international meta-analysis showed that higher mortality was strongly associated with increasing age, male gender and the presence of obesity, HT, DM, CVD, and cancer, and with some excess mortality associated with several other comorbidities [20]. Other studies in the Middle East examined the risk of disease severity or of severe complications. In addition to increasing age and male gender, these studies identified as risk factors, CKD [21, 22] and cancer [21], but also DM and/or obesity [10, 21–23], HT and/or CHD/CVD [21–23]. Two studies in Qatar and Kuwait, specifically focusing on ICU admission, stressed the increased risk associated with DM [24], in contrast with our results, findings in the literature are quite heterogeneous. Actually, meta-analyses found that DM was associated with higher risk of composite event combining ICU admission with mortality and/or severe/critical form of the disease [25–27], while other meta-analyses found an absence of increased ICU risk [28, 29] or the evidence inconclusive [30]. Furthermore, to our knowledge, most studies did not account for the time component in their analysis and none used a flexible model.

Our stratified analysis reinforces the evidence about differences in COVID-19 outcomes between nationals and non-nationals in Kuwait [31] (please see Section 4 in the Appendix). These differences may be attributed to disparities in the socio-economic status between resident citizens and migrant workers, an observation which has also been noted in other populations with large migrant worker population [32–34]. Migrant workers in the Gulf countries are also predominantly of South Asian descent, as opposed to the citizen resident populations, which are largely of Middle Eastern origin [35]. Large prospective cohort studies [36, 37] found that ethnic minorities with COVID-19 were more likely to be admitted to critical care, particularly South Asians who had a higher mortality rate, compared to other ethnicities. Evidence about the role played by genetic variation remains weak [38]. Our models however revealed a much stronger cohort effect on non-Kuwaiti with respect to ICU admission either due to many poor prognosis cases after 12th May or many asymptomatic cases before 12th May compared to Kuwaiti. Higher hazards of ICU admission were observed in non-Kuwaiti with asthma/COPD and CKD and in Kuwaiti with CVD/HT and weakened immune system.

In conclusion, the results of this study provided useful insight in describing the probability of ICU admission and hospital discharge according to age, gender and comorbidities of confirmed COVID-19 cases in Kuwait. The probabilities provided a deeper understanding of this demand according to someone’s characteristics which is essential to hospital management. Notably, the design of the study allowed for the estimation of ‘real-world probabilities’ [39] avoiding hypothetical scenarios and convoluted interpretations [40]. Further work is needed to build predictive models which will help to improve the management of the COVID-19 pandemic and the patients.

## Supplementary Information

**Additional file 1** Appendix.

## Data Availability

The dataset used for the current study is not publicly available due to the Ministry of Health protection from possible recognition of a patient. To guarantee the confidentiality of personal and health information, only the authors have had access to the data during the study in accordance with the relevant licence agreements. For access of data, please contact: Dr. Sarah Al-Youha: sarahalyouha@gmail.com

## References

[1] Huang C, Wang Y, Li X, Ren L, Zhao J, Hu Y, Zhang L, Fan G, Xu J, Gu X (2020). Clinical features of patients infected with 2019 novel coronavirus in Wuhan, China. Lancet.

[2] World Health Organization. Naming the coronavirus disease (COVID-19) and the virus that causes it. 2020. (Accessed 29 Oct 2020). https://www.who.int/emergencies/diseases/novel-coronavirus-2019/technical-guidance/naming-the-coronavirus-disease-(covid-2019)-and-the-virus-that-causes-it.

[3] Van Damme W, Dahake R, Delamou A, Ingelbeen B, Wouters E, Vanham G, Van De Pas R, Dossou J-P, Ir P, Abimbola S (2020). The COVID-19 pandemic: diverse contexts; different epidemics–how and why?. BMJ Global Health.

[4] Wu F, Zhao S, Yu B, Chen Y-M, Wang W, Song Z-G, Hu Y, Tao Z-W, Tian J-H, Pei Y-Y (2020). A new coronavirus associated with human respiratory disease in China. Nature.

[5] World Health Organization. Timeline: WHO’s COVID-19 response. 2020. (Accessed 29 Oct 2020). https://www.who.int/emergencies/diseases/novel-coronavirus-2019/interactive-timeline#event-42.

[6] World Health Organization. Coronavirus disease (COVID-10) pandemic, Number at a glance. 2020. (Accessed 29 Oct 2020). https://www.who.int/emergencies/diseases/novel-coronavirus-2019/interactive-timeline#event-42.

[7] Alabdulkarim N, Alsultan F, Bashir S (2020). Gulf countries responding to COVID-19. Dubai Med J.

[8] Alandijany TA, Faizo AA, Azhar EI (2020). Coronavirus disease of 2019 (COVID-19) in the Gulf Cooperation Council (GCC) countries: Current status and management practices. J Infect Public Health.

[9] Almazeedi S, Al-Youha S, Jamal MH, Al-Haddad M, Al-Muhaini A, Al-Ghimlas F, Al-Sabah S. Characteristics, risk factors and outcomes among the first consecutive 1096 patients diagnosed with COVID-19 in Kuwait. EClinicalMedicine. 2020:100448. 10.1016/j.eclinm.2020.100448 PMC7335246 32766546

[10] Al-Shammari AA, Ali H, Alahmad B, Al-Refaei FH, Al-Sabah S, Jamal MH, Alshukry A, Al-Duwairi Q, Al-Mulla F. COVID-19 transmission and forecasting in Kuwait: A mathematical modeling study. 2020. Available at SSRN: https://ssrn.com/abstract=3618104 or 10.2139/ssrn.361804.

[11] Alkhamis MA, Al Youha S, Khajah MM, Haider NB, Alhardan S, Nabeel A, Al Mazeedi S, Al-Sabah SK (2020). Spatiotemporal dynamics of the COVID-19 pandemic in the State of Kuwait. Int J Infect Dis.

[12] Charvat H, Remontet L, Bossard N, Roche L, Dejardin O, Rachet B, Launoy G, Belot A (2016). A multilevel excess hazard model to estimate net survival on hierarchical data allowing for non-linear and non-proportional effects of covariates. Stat Med.

[13] Sauerbrei W, Perperoglou A, Schmid M, Abrahamowicz M, Becher H, Binder H, Dunkler D, Harrell FE, Royston P, Heinze G (2020). State of the art in selection of variables and functional forms in multivariable analysis–outstanding issues. Diagn Prognostic Res.

[14] Kipourou D-K, Charvat H, Rachet B, Belot A (2019). Estimation of the adjusted cause-specific cumulative probability using flexible regression models for the cause-specific hazards. Stat Med.

[15] Griffith GJ, Morris TT, Tudball MJ (2020). Collider bias undermines our understanding of COVID-19 disease risk and severity. Nat Commun.

[16] Abohamr SI, Abazid RM, Aldossari MA, Amer HA, Badhawi OS, Aljunaidi OM, Alzarzour SH, Saadeddin HM, Bhat FA, Elsheikh E (2020). Clinical characteristics and in-hospital mortality of COVID-19 adult patients in Saudi Arabia. Saudi Med J.

[17] Alamdari NM, Afaghi S, Rahimi FS, Tarki FE, Tavana S, Zali A, Fathi M, Besharat S, Bagheri L, Pourmotahari F (2020). Mortality risk factors among hospitalized COVID-19 patients in a major referral center in Iran. Tohoku J Exp Med.

[18] Khamis F, Al-Zakwani I, Al Naamani H, Al Lawati S, Pandak N, Omar MB, Al Bahrani M, Bulushi ZAL, Al Khalili H, Al Salmi I (2020). Clinical characteristics and outcomes of the first 63 adult patients hospitalized with COVID-19: an experience from Oman. J Infect Public Health.

[19] Nikpouraghdam M, Farahani AJ, Alishiri G (2020). Epidemiological characteristics of coronavirus disease 2019 (COVID-19) patients in IRAN: A single center study. J Clin Virol.

[20] Noor FM, Islam MM (2020). Prevalence and associated risk factors of mortality among COVID-19 patients: A meta-analysis. J Community Health.

[21] Radwan NM, Mahmoud NE, Alfaifi AH, Alabdulkareem KI (2020). Comorbidities and severity of coronavirus disease 2019 patients. Saudi Med J.

[22] Yanover C, Mizrahi B, Kalkstein N, Marcus K, Akiva P, Barer Y, Shalev V, Chodick G (2020). What factors increase the risk of complications in SARS-CoV-2–infected patients? a cohort study in a Nationwide Israeli Health Organization. JMIR Public Health Surveill.

[23] Al Kuwari HM, Abdul Rahim HF, Abu-Raddad LJ, Abou-Samra AB, Al Kanaani Z, Al Khal A, Al Kuwari E, Al Marri S, Al Masalmani M, Al Romaihi HE (2020). Epidemiological investigation of the first 5685 cases of SARS-CoV-2 infection in Qatar, 28 February–18 April 2020. BMJ Open.

[24] Omrani AS, Almaslamani MA, Daghfal J, Alattar RA, Elgara M, Shaar SH, Ibrahim TBH, Zaqout A, Bakdach D, Akkari AM (2020). The first consecutive 5000 patients with coronavirus disease 2019 from Qatar; a nation-wide cohort study. BMC Infect Dis.

[25] Nandy K, Salunke A, Pathak SK, Pandey A, Doctor C, Puj K, Sharma M, Jain A, Warikoo V (2020). Coronavirus disease (COVID-19): A systematic review and meta-analysis to evaluate the impact of various comorbidities on serious events. Diabetes Metab Syndr: Clin Res Rev.

[26] Zhou Y, Yang Q, Chi J (2020). Comorbidities and the risk of severe or fatal outcomes associated with coronavirus disease 2019: A systematic review and meta-analysis. Int J Infect Dis.

[27] Figliozzi S, Masci PG, Ahmadi N, Tondi L, Koutli E, Aimo A, Stamatelopoulos K, Dimopoulos MA, Caforio ALP, Georgiopoulos G (2020). Predictors of adverse prognosis in COVID-19: A systematic review and meta-analysis. Eur J Clin Investig.

[28] Parveen R, Sehar N, Bajpai R, Agarwal NB (2020). Association of diabetes and hypertension with disease severity in COVID-19 patients: A systematic literature review and exploratory meta-analysis. Diabetes Res Clin Pract.

[29] Zhao J, Li X, Gao Y, Huang W (2020). Risk factors for the exacerbation of patients with 2019 novel coronavirus: A meta-analysis. Int J Med Sci.

[30] Sales-Peres SHC, de Azevedo-Silva LJ, Bonato RCS, Sales-Peres MC, Pinto ACDS, Santiago Junior JF (2020). Coronavirus (SARS-CoV-2) and the risk of obesity for critically illness and ICU admitted: Meta-analysis of the epidemiological evidence. Obes Res Clin Pract.

[31] Hamadah H, Alahmad B, Behbehani M, Al-Youha S, Almazeedi S, Al-Haddad M, Jamal MH, Al-Sabah S (2020). COVID-19 clinical outcomes and nationality: results from a nationwide registry in Kuwait. BMC Public Health.

[32] Pham KT, Nguyen LH, Vuong Q-H, Ho M-T, Vuong T-T, Vu GT, Nguyen HLT, Tran BX, Latkin CA, Ho CSH (2019). Health inequality between migrant and non-migrant workers in an industrial zone of Vietnam. Int J Env Res Public Health.

[33] Pradhan B, Kjellstrom T, Atar D, Sharma P, Kayastha B, Bhandari G, Pradhan PK (2019). Heat stress impacts on cardiac mortality in Nepali migrant workers in Qatar. Cardiology.

[34] Mishra SR, Ghimire S, Joshi C, Gyawali B, Shrestha A, Neupane D, Sharma SR, Pokharel Y, Virani SS (2019). Cardio-metabolic disease risk factors among South Asian labour migrants to the Middle East: a scoping review and policy analysis. Glob Health.

[35] Chaabna K, Cheema S, Mamtani R (2017). Migrants, healthy worker effect, and mortality trends in the Gulf Cooperation Council countries. PloS ONE.

[36] Harrison EM, Docherty AB, Barr B, Buchan I, Carson G, Drake TM, Dunning J, Fairfield CJ, Gamble C, Green CA, et al. Ethnicity and outcomes from COVID-19: the ISARIC CCP-UK prospective observational cohort study of hospitalised patients. 2020. Available at SSRN: https://ssrn.com/abstract=3618215 or 10.2139/ssrn.3618215.

[37] Williamson EJ, Walker AJ, Bhaskaran K (2020). Factors associated with COVID-19-related death using OpenSAFELY. Nature.

[38] Khunti K, Singh AK, Pareek M, Hanif W (2020). Is ethnicity linked to incidence or outcomes of COVID-19?. BMJ.

[39] Charvat H, Bossard N, Daubisse L, Binder F, Belot A, Remontet L (2013). Probabilities of dying from cancer and other causes in French cancer patients based on an unbiased estimator of net survival: a study of five common cancers. Cancer Epidemiol.

[40] Austin PC, Fine JP (2017). Practical recommendations for reporting Fine-Gray model analyses for competing risk data. Stat Med.

