# Correction to: Probabilities of ICU admission and hospital discharge according to patient characteristics in the designated COVID-19 hospital of Kuwait

**DOI:** 10.1186/s12889-021-11146-4

**Published:** 2021-06-22

**Authors:** Dimitra-Kleio Kipourou, Clémence Leyrat, Nourah Alsheridah, Sulaiman Almazeedi, Sarah Al-Youha, Mohammad H. Jamal, Mohannad Al-Haddad, Salman Al-Sabah, Bernard Rachet, Aurélien Belot

**Affiliations:** 1 Inequalities in Cancer Outcomes Network, Department of Non-Communicable Disease Epidemiology, Faculty of Epidemiology and Population Health, London School of Hygiene & Tropical Medicine, London, UK; 2 Department of Medical Statistics, Faculty of Epidemiology and Population Health, London School of Hygiene & Tropical Medicine, Keppel Street, London, UK; 3 COVID-19 Research Group, Jaber Al-Ahmad Al-Sabah Hospital, Kuwait City, Kuwait

**Correction to: BMC Public Health 21, 799 (2021)**

**https://doi.org/10.1186/s12889-021-10759-z**

It was highlighted that in the original article [1] the Additional file 1 was missing. This Correction article includes the Additional file 1. The original article has been updated.

## Supplementary Information

**Additional file 1.**
