# Supplementary material for: Probabilities of ICU admission and hospital discharge according to patient characteristics in the designated COVID-19 hospital of Kuwait
Source: BMC Public Health. 2021 Apr 26;21:799. doi: 10.1186/s12889-021-10759-z (PMC8072736; doi:10.1186/s12889-021-10759-z)
Supplement: Supplementary file 1 — Additional file 1 Appendix. [file 12889_2021_10759_MOESM1_ESM.pdf]

Appendix of the paper entitled ”Probabilities of ICU admission and hospital discharge according to patient characteristics in the designated COVID-19 hospital of Kuwait”

Dimitra-Kleio Kipourou[1*], Clémence Leyrat[2,1], Nourah Alsheridah[1], Sulaiman Almazeedi[3], Sarah Al-Youha[3], Mohammad H. Jamal[3], Mohannad Al-Haddad[3], Salman Al-Sabah[3], Bernard Rachet[1], Aurélien Belot[1]

[1] Inequalities in Cancer Outcomes Network, Department of Non-Communicable Disease Epidemiology,Faculty of Epidemiology and Population Health, London School of Hygiene & Tropical Medicine
[2] Department of Medical Statistics, Faculty of Epidemiology and Population Health, London School of Hygiene & Tropical Medicine
[3] COVID-19 Research Group, Jaber Al-Ahmad Al-Sabah Hospital, Kuwait

*dimitra-kleio.kipourou@lshtm.ac.uk

Abbreviations: competing risks; COVID-19; probability of ICU admission

S1.1 Event-specific cumulative probabilities

The cumulative progression over time to the event of interest is known as the cause-specific cumulative probability $F_j(t)$ and it is defined as the probability of progressing to event j in the presence of other events between time 0 and t as a function of time since diagnosis

$$F_j(t) = Pr[0 \leq T \leq t, J = j]$$

This quantity is determined by all event - specific hazards hence, the probability of event j is defined as

$$F_j(t) = P(T \leq t, event = j) \int_0^t \lambda_j(u) S(u) du \tag{1}$$

where $S(t)$ is the overall survival, $S(t) = \exp\left(-\int_0^t \sum_{j=1}^{J} \lambda_j(u) du\right)$ [1, 2].

Non-Parametric estimation of probabilities

We can use the Aalen - Johansen estimator for the non - parametric estimation of the event - specific cumulative probabilities as follows

$$\hat{F}_j(t) = \int_0^t \hat{S}_{\text{KM}}(u-) d\hat{\lambda}_j(u) \tag{2}$$

where $\hat{S}_{\text{KM}}$ is the Kaplan-Meier estimator of the overall survival and $d\hat{\lambda}_j(u)$ is the increment of the Nelson-Aalen estimator.

With this estimator we are able to compute the cumulative probability of a specific event for a population or a subgroup but not for individuals.

Estimation with Flexible Regression Models (FRM)

Conversely, using a modelling approach we are able to predict for specific covariate combinations allowing for individual (and population) predictions. Here, we employ an FRM for the (logarithm of) the (event j)-specific hazard expressed as

$$\log[\lambda_j(t, \mathbf{x}; \boldsymbol{\beta}_j)] = \log(\lambda_0(t; \boldsymbol{\gamma}_j)) + \mathbf{x}^\top \boldsymbol{\alpha}_j(t)$$

where $\boldsymbol{\beta}_j$ is a vector of regression parameters used which includes the parameters for (i) the baseline hazard and (ii) the time-dependent (cause j)-specific hazard ratios, *i.e.* $\boldsymbol{\beta}_j = (\boldsymbol{\gamma}_j^\top, \boldsymbol{\alpha}_j^\top)^\top$. This type of model uses B-spline functions to model the logarithm of the baseline hazard parameters $\boldsymbol{\gamma}_j$ and the time-dependent (event j)-specific hazard ratios $\boldsymbol{\alpha}_j(t)$ for the corresponding vector of covariates $\mathbf{x}$.

For the estimation of the probabilities we should fit as many models as distinct events of interest and further combine the results as follows

$$\hat{F}_j(t, \mathbf{x}_i; \hat{\boldsymbol{\beta}}) = \int_0^t \hat{S}(u, \mathbf{x}_i; \hat{\boldsymbol{\beta}}) \hat{\lambda}_j(u, \mathbf{x}_i; \hat{\boldsymbol{\beta}}_j) du \tag{3}$$

For more details the reader is referred to the paper of Kipourou et al. [3].

Confidence intervals

The approximate $100 * (1 - \alpha)\%$ confidence interval of $\hat{F}_j(t, \mathbf{x}; \hat{\boldsymbol{\beta}})$ is based on the (approximate) normality assumption on the complementary log - log scale thus we can estimate the

$$\hat{F}_j(t, \mathbf{x}; \hat{\boldsymbol{\beta}})^\Omega \tag{4}$$

where $\Omega = \left\{\exp\left\{\pm z_\alpha \text{s.e.}\left[\log\left(-\log(\hat{F}_j(t, \mathbf{x}; \hat{\boldsymbol{\beta}}))\right)\right]\right\}\right\}$, z_α is the (1-$\alpha/2$) quantile of the standard normal distribution.

$$\text{Var}\left[\log\left(-\log(\hat{F}_j(t, \mathbf{x}; \hat{\boldsymbol{\beta}}))\right)\right] = \frac{\text{Var}[\hat{F}_j(t, \mathbf{x}; \hat{\boldsymbol{\beta}})]}{\left(\log(\hat{F}_j(t, \mathbf{x}; \hat{\boldsymbol{\beta}}))(\hat{F}_j(t, \mathbf{x}; \hat{\boldsymbol{\beta}}))\right)^2} \tag{5}$$

We could also use the $1 - \hat{F}_j(t, \mathbf{x}; \hat{\boldsymbol{\beta}})$ instead to avoid issues with the denominator.

S1.2 Non - parametric cumulative probabilities of ICU admission and discharge

Fig. 1: Non-parametric estimates of the probabilities of ICU admission and hospital discharge for the whole population followed by a table showing the number of individuals at risk and the cumulative number of each type of event.

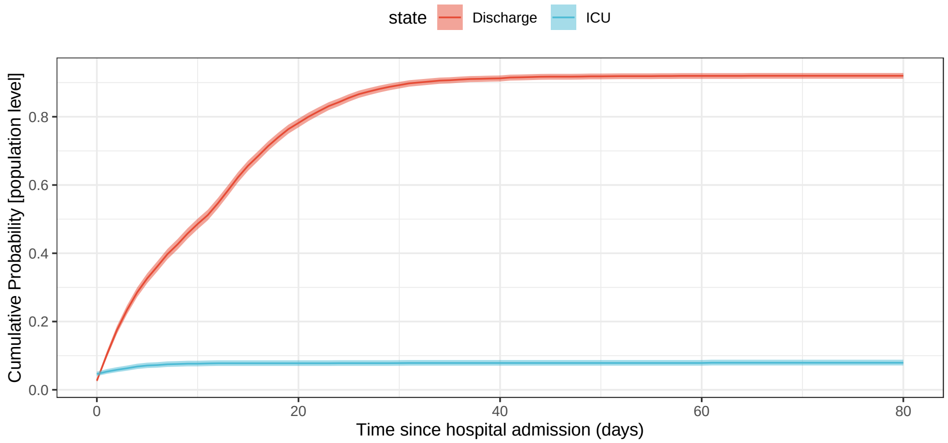

	0	1	5	10	20	30	40	50	60	70	80
At risk	3995	3365	2386	1722	544	112	33	9	3	2	0
Censored	0	3	23	36	51	53	57	59	60	60	61
ICU	0	213	284	305	310	312	313	313	315	315	315
Discharged	0	414	1302	1932	3090	3518	3592	3614	3617	3618	3619

S1.3 Model selection

Table 1: Results from FRM for the event-specific hazard of ICU admission. Results show the variables in the models and their respective AIC and BIC. Mod1 includes the quadratic spline terms for age and the binary variables: admission date before or after the 12th of May 2020, gender, chronic kidney disease (CKD), cardiovascular diseases (CVD) or hypertension (HT), asthma or chronic obstructive pulmonary disease (COPD), weakened immune system and other comorbidities such as dyslipidemia, hepatitis, hypothiroidism, recent surgery (during the past 30 days) etc. Results are sorted based on BIC.

	AIC	BIC
Mod1	2272.10	2348.15
Mod1 + interaction (age x Admission date ≥ 12/05/2020)	2265.13	2355.43
Mod1 + interaction (age x CVD/HT)	2265.35	2355.65
Mod1 + interaction (age x Gender)	2266.26	2356.56
Mod1 + interaction (age x Other)	2266.60	2356.90
Mod1 + interaction (age x Asthma/COPD)	2272.55	2362.85
Mod1 + interaction (age x CKD)	2273.37	2363.67
Mod1 + interaction (age x Weakened immune system)	2273.54	2363.84
Mod1 + interaction(age x Diabetes)	2273.71	2364.01

Table 2: Results from FRM for the event-specific hazard of discharge. Mod1 includes the quadratic spline terms for age and the binary variables: admission date before or after the 12th of May 2020, gender, chronic kidney disease (CKD), cardiovascular diseases (CVD) or hypertension (HT), asthma or chronic obstructive pulmonary disease (COPD), weakened immune system and other comorbidities such as dyslipidemia, hepatitis, hypothiroidism, recent surgery (during the past 30 days) etc. Results are sorted based on BIC.

	AIC	BIC
Mod1 + interaction (age x Gender)	24179.04	24315.73
Mod1	24214.07	24329.17
Mod1 + interaction(age x Other)	24208.18	24344.86
Mod1 + interaction(age x Admission>= 12/05/2020)	24209.07	24345.75
Mod1 + interaction(age x CKD)	24209.11	24345.80
Mod1 + interaction(age x Diabetes)	24210.08	24346.77
Mod1 + interaction(age x Weakened immune system)	24210.63	24347.31
Mod1 + interaction(age x CVD/HT)	24211.27	24347.95
Mod1 + interaction(age x Asthma/COPD)	24215.63	24352.31

Fig. 2: Comparison of model-based vs. non-parametric population cumulative probability event-specific estimates as an indirect way of assessing the performance of the models.

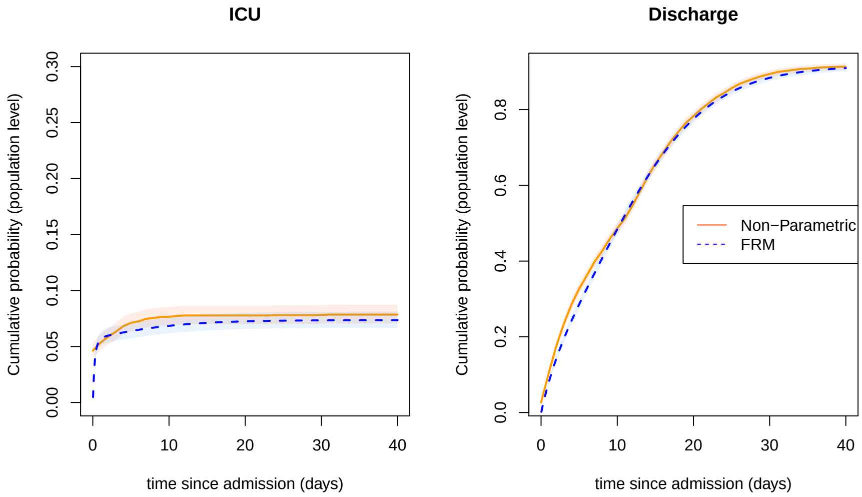

S1.4 Event-specific hazard ratios

Fig. 3: ICU - specific hazard ratios with confidence intervals based on the full cohort (using Cox and FRM) and separately for Kuwaiti and non - Kuwaiti population (using FRM).

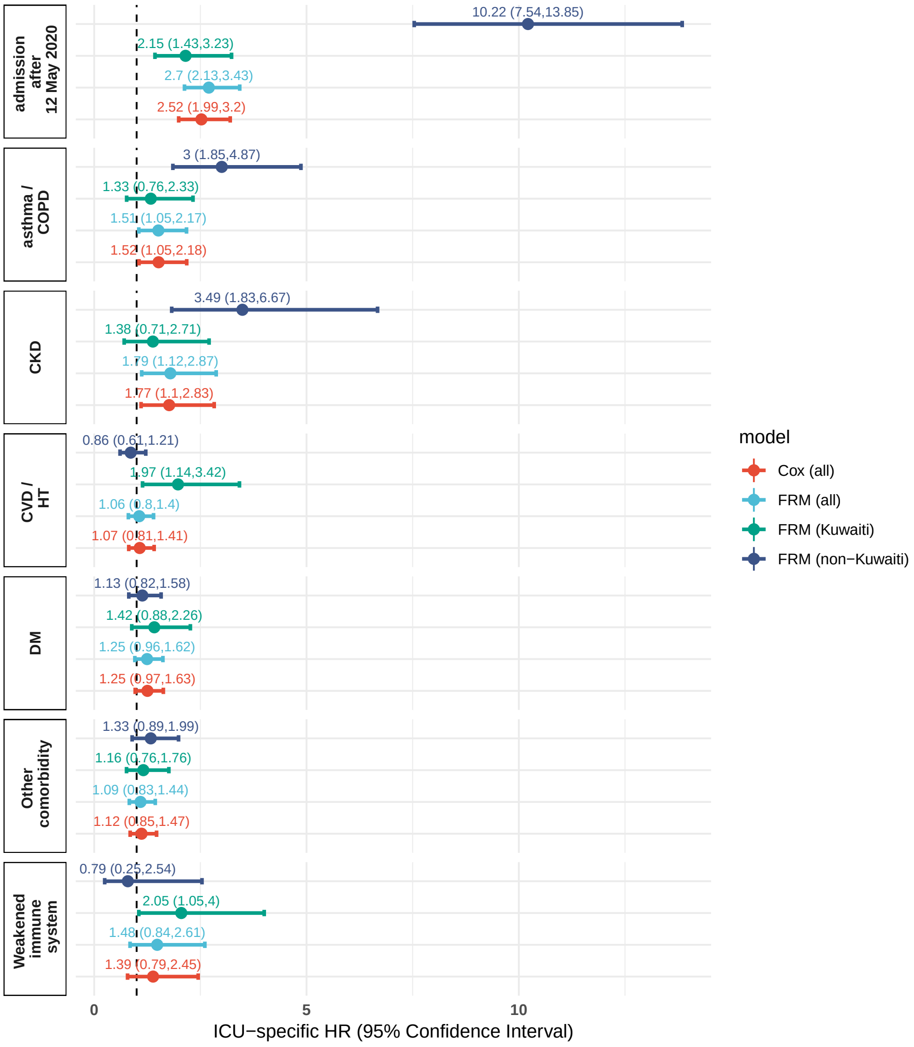

Fig. 4: Discharge - specific hazard ratios with confidence intervals based on the full cohort (using Cox and FRM) and separately for Kuwaiti and non - Kuwaiti population (using FRM).

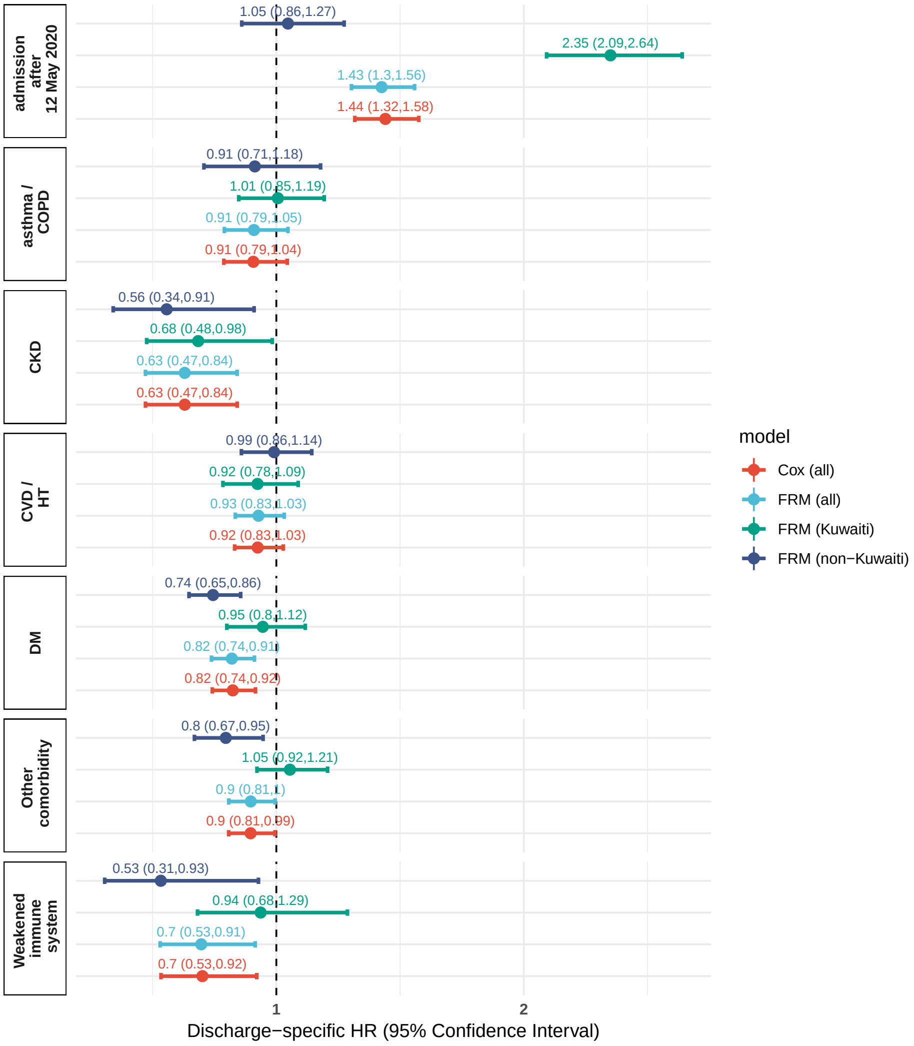

S1.5 Event-specific probabilities estimated for the period before May 12th, 2020

Fig. 5: Cumulative probability of ICU admission according to baseline characteristics (age, gender and existence of comorbidities) for females (upper row) and males (lower row) predicted within 1, 5 and 10 days post hospital admission. Comorbidities include: chronic kidney disease (CKD), cardiovascular diseases (CVD) or hypertension (HT), asthma or chronic obstructive pulmonary disease (COPD), weakened immune system and other comorbidities such as dyslipidemia, hepatitis, hypothiroidism, recent surgery (during the past 30 days) etc.

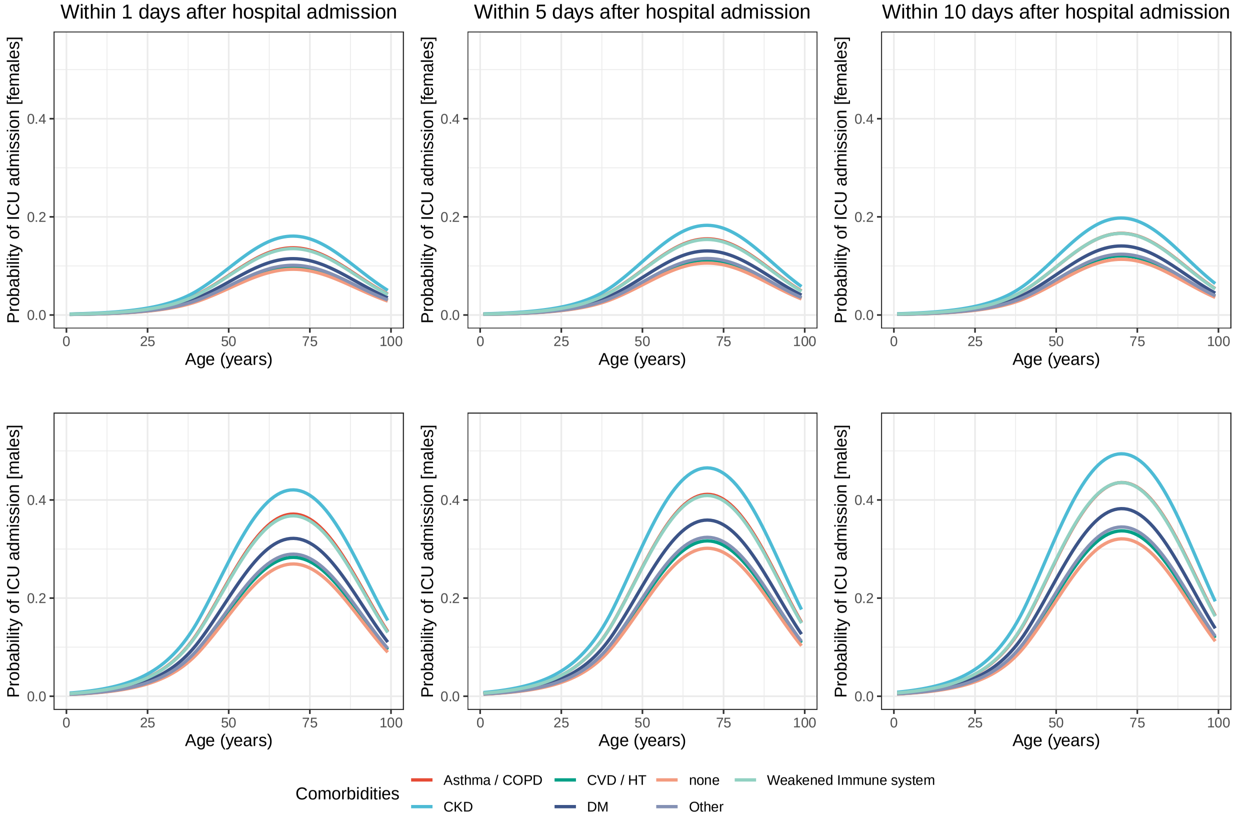

Fig. 6: Cumulative probability of hospital discharge according to baseline characteristics (age, gender and existence of comorbidities) for females (upper row) and males (lower row) predicted within 10, 20 and 30 days post hospital admission. Comorbidities include: chronic kidney disease (CKD), cardiovascular diseases (CVD) or hypertension (HT), asthma or chronic obstructive pulmonary disease (COPD), weakened immune system and other comorbidities such as dyslipidemia, hepatitis, hypothiroidism, recent surgery (during the past 30 days) etc.

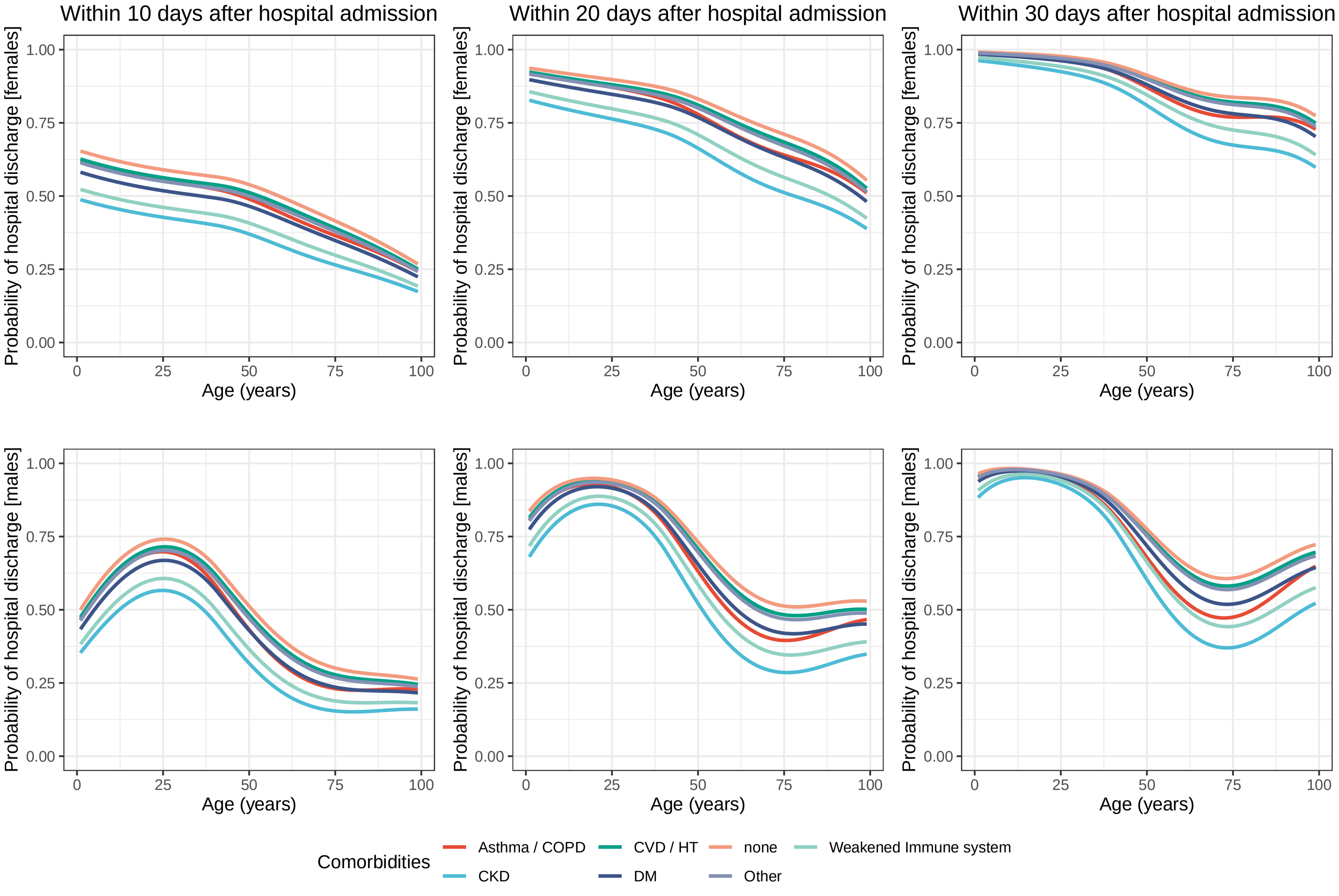

References

[1] Ruth M Pfeiffer and Mitchell H Gail. *Absolute risk: methods and applications in clinical management and public health*. CRC Press, 2017.

[2] RB Geskus. *Data analysis with competing risks and intermediate states*, volume 82. CRC Press, 2015.

[3] D-K Kipourou, H Charvat, B Rachet, and A Belot. Estimation of the adjusted cause-specific cumulative probability using flexible regression models for the cause-specific hazards. *Statistics in medicine*, 38(20):3896–3910, 2019.
